# Erectile Dysfunction in Tunisian Veterans: Clinical and Psychological Insights

**DOI:** 10.1192/j.eurpsy.2025.2294

**Published:** 2025-08-26

**Authors:** Y. Ben Othmene, A. Baatout, W. Kabtni, K. Kefi, S. Eddif, H. Kefi, A. Oumaya

**Affiliations:** 1 Psychiatry, Military Hospital, Tunis, Tunisia

## Abstract

**Introduction:**

Erectile dysfunction (ED) is a multifactorial condition affecting a significant propotion of men worldwide, impacting their mental well-being, life quality and interpersonal relationships. Often associated with various underlying factors, ED manifests differently in each individual, yet there is a limited understanding of the unique profiles of specific populations, such as military veterans, who may face distinct physical, psychological and social challenges contributing to the condition.

**Objectives:**

This study aimed to explore the clinical profile, demographic factors, and associated comorbidities of Tunisian military veterans affected by ED.

**Methods:**

A cross-sectional descriptive survey was conducted between September and November 2024 on Tunisian veterans seeking consult, using a data file and 2 self-report scales:

The Hospital Anxiety and Depression (HAD) scale, consisting of two subscales: Anxiety (A) and Depression (D), dividing patients into: normal[0-7], borderline case[8-10], an abnormal level of anxiety or depression[11-21].

The IIEF-5 (International Index of Erectile Function 5) to evaluate ED with six categories: [1-4]: uninterpretable, [5-7]: severe, [8-11]: moderate, [12-16]: mild to moderate, [17-21]: mild and [22-25]: no ED.

**Results:**

The study enrolled 28 veterans diagnosed with ED, with an average age of 40 [25-61] years. Most (78.6%) were married while 17.9% were single and 3.6% were divorced.

Smoking was prevalent in 82.1% and 21.4% reported alcohol consumption, with 83.3% drinking occasionally and 16.6% drinking regularly. None reported using cannabis or other illicit drugs.

Regarding medical history, 39.3% had medical health conditions including varicocele, diabetes, combined arterial hypertension and diabetes, myocardial infarction and other health issues.

Psychiatric follow-up was reported by 85.7% of the individuals.

Anxiety symptoms were present in 89.3% of participants with 3.6% classified as borderline cases. Depressive symptoms were reported by 67.9% with 21.4% categorized as borderline symptoms.

Notably, 64.3% of the veterans experienced both anxiety and depressive symptoms and were taking antidepressants, while only one veteran exhibited neither. The remaining had either anxiety or depressive symptoms.

In terms of ED severity, 46.4% had mild to moderate ED, followed by 28.6% with mild ED, 17.9% with severe ED and 7.1% with moderate ED.

Only 14.3% reported using sexual enhancers.

**Conclusions:**

This study provides valuable insights into the unique profile of ED among Tunisian veterans, revealing a high prevalence of psychological comorbidities, particularly anxiety and depression, alongside physical health issues. The interconnection of these factors highlights the importance of a holistic approach adressing both psychological and physical aspects of ED. These results call for further research into the specific challenges faced by veterans for a more personalized and effective interventions.

**Disclosure of Interest:**

None Declared

